# Colonic Transit Time and Gut Peptides in Adult Patients with Slow and Normal Colonic Transit Constipation

**DOI:** 10.1155/2017/3178263

**Published:** 2017-09-07

**Authors:** Giuseppe Riezzo, Guglielmina Chimienti, Caterina Clemente, Benedetta D'Attoma, Antonella Orlando, Caterina Mammone Rinaldi, Francesco Russo

**Affiliations:** ^1^Laboratory of Nutritional Pathophysiology, National Institute of Digestive Diseases, IRCCS “Saverio de Bellis”, Castellana Grotte, Bari, Italy; ^2^Department of Biosciences, Biotechnology and Biopharmaceutics, University of Bari, Bari, Italy; ^3^Department of Radiology and Imaging, National Institute of Digestive Diseases, IRCCS “Saverio de Bellis”, Castellana Grotte, Bari, Italy

## Abstract

**Purpose:**

To investigate whether pathophysiological differences exist among healthy controls (HC) and patients with slow and normal transit constipation (STC and NTC), we evaluated (1) gastrointestinal (GI) symptoms using validated questionnaires; (2) circulating concentrations of neurotensin, motilin, corticotrophin-releasing factor (CRF), and somatostatin; and (3) possible differences in frequency distribution of the neurotensin rs1800832 A/G and Neurotensin Receptor 1 rs6090453 C/G SNPs.

**Methods:**

Fifty-one patients with severe functional constipation and 20 HC completed the study. Symptoms were evaluated by GSRS and Constipaq scoring system. Plasma concentrations of GI peptides were evaluated by ELISA on fasting and six sequential blood samples after a standard meal. Genotyping was performed by PCR and endonuclease digestion.

**Results:**

Symptom profiles largely overlapped between NTC and STC patients. As for peptide profiles, neurotensin showed lower concentrations at 60 and 90 min in STC versus HC, and motilin showed throughout the curve 85% and 82% lower levels in STC than HC and NTC, respectively. Finally, neurotensin polymorphism resulted in being associated with the peptide levels.

**Conclusions:**

Symptom profile is not a reliable tool to discriminate STC, whilst the GI peptide profiles might help in identifying it.

## 1. Introduction

Functional constipation is a multifactorial disease very frequent among Europeans with an estimated prevalence of 17.1% [1]. In order to obtain a standardized definition of functional constipation, the Rome criteria (Rome I–IV) [2] have been developed and proposed, even though they are currently used for research purposes, mainly in clinical trials.

Functional constipation is thought to be the result of several causes including diet low in fibre content, autonomic neuropathy, disorders of the enteric nervous system, and alterations in the profile of circulating gastrointestinal (GI) peptides. A proposed classification of constipation according to the colonic transit time categorized patients as having* “slow transit constipation”* (STC) or* “normal transit constipation”* (NTC) [3]. STC seems to be caused by impaired colonic motility; even if it has also been observed that dysmotility disorders in these patients are not limited to the gut but, rather, they extend to other GI tracts such as esophagus, stomach, small bowel, gallbladder, and anorectum [4]. These data have suggested the involvement of a dysfunctional enteric nervous system, as already proposed as a cause of STC in subjects who have undergone pelvic surgery or have given birth [5]. The study of the neuroendocrine profile in STC patients had shown that a number of gut peptides, including motilin, corticotrophin-releasing factor (CRF), somatostatin, and neurotensin, may induce alterations of colonic motility [6–8]. In rabbits, motilin has been demonstrated to excite the colon through pathways different from those of cholinergic motor nerves [9] and, more recently, a research performed in humans described lower serum motilin levels in constipated children than healthy subjects [10]. CRF signalling induced by stress is well established to delay gastric emptying and stimulate colonic functions [11], and somatostatin receptors are expressed in human circular and longitudinal colonic smooth muscle cells [12]. As for neurotensin, beyond its known role as stimulating gut motility [13], this peptide has also profound opioid-independent analgesic effects and this aspect could have interesting pathophysiological implications for symptoms of constipation [14].

Recently, a possible familial clustering of functional constipation has been proposed in children [15]; consequently, it is conceivable that genetics may play a part in the development of the disease. Besides, many efforts have been made to determine genetic factors involved in the pathogenesis of the irritable bowel syndrome (IBS), a common GI functional disorder characterized by altered bowel habits. Various polymorphisms have been investigated in search for causality, considering as candidate genes those coding for neurotransmitters and related molecules, inflammation mediators, and GI peptides, however, without firm conclusions [16]. As to a genetic component in functional constipation, two motilin polymorphisms have been recently investigated in a paediatric population, without significant differences between constipated patients and healthy subjects [10]. The polymorphic proneurotensin gene* (NTS)* could be an interesting candidate, due to the multiple roles played by neurotensin as local hormone and neurotransmitter in the enteric nervous system [17].

*NTS* variants, along with polymorphisms in the Neurotensin Receptor 1* (NTR1)* gene coding for a high affinity neurotensin receptor, have already been investigated for their possible roles in neuropsychiatric disorders [18], but no data concerning neurotensin system genetics and constipation are available in literature. A previously published research by our group highlighted that the* NTS* rs1800832 A/G SNP was associated with circulating levels of the peptide in patients with functional dyspepsia, letting us hypothesize a possible role for neurotensin genetics in the symptom profile of the disease [19].

In order to improve the characterization of functional constipation and to assess whether pathophysiological differences exist among HC, STC, and NTC patients, aims of the present study were to (1) analyse the symptom profile using validated questionnaires in STC and NTC patients; (2) evaluate the circulating concentrations of neurotensin, motilin, CRF, and somatostatin; (3) establish whether the frequencies of two polymorphisms of the neurotensin system genes, namely, the* NTS* rs1800832 A/G in the 5′ untranslated region (UTR) and the intron variant rs6090453 C/G in the* NTR1* gene, differ between healthy subjects and STC and NTC patients and whether these polymorphisms are associated with the circulating concentrations of neurotensin.

## 2. Methodology

### 2.1. Clinical Evaluation

Eighty patients with functional constipation were recruited from the outpatients of the National Institute of Digestive Diseases, IRCCS* “Saverio de Bellis”*, Castellana Grotte, Bari, Italy, from March 2011 to February 2015.

The inclusion criteria were as follows: fulfilment of the Rome III criteria for functional constipation [20], availability of at least one GI imaging study during the last 5 years (colonoscopy, sigmoidoscopy, abdominal ultrasound, and barium enema), and age of 19–70 years. The exclusion criteria were IBS including the IBS-constipation subtype, organic constipation, intake of drugs, metabolic diseases, GI diseases, diseases of the enteric nervous system/muscle, concomitant participation in other clinical trials, ingestion of probiotics/prebiotics less than two weeks before the inclusion in the study, major GI surgery, pregnancy, family history of cancer or inflammatory bowel disease, blood disorders, impaired thyroid function, and recent trips to countries with endemic parasitic diseases. Written informed consent was obtained from all participants.

The study design provided a preliminary visit* (visit 1)* to sign the informed consent and to receive the symptom diary to be completed at home. The same day, the patient data and questionnaires related to GI symptoms were completed and a physical examination was performed. At* visit 2*, after a period of 7 days during which the patients had to avoid the use of laxatives or enemas, the same clinical evaluations were performed and the calculation of the time of the colonic transit was completed. Besides blood samples were collected for subsequent biochemical and genetic assessments.

Healthy controls (HC) were recruited for GI peptide evaluations within the administrative staff of our institute after they were interviewed and gave their written informed consent. Subjects were excluded if they had a body mass index (kg/m^2^) >25, had a significant current or previous medical history, were regularly taking medication (other than oral contraceptives) that may have affected the GI or central nervous system, consumed >20 units alcohol/wk., smoked, or had donated blood within the 4–6 months before their inclusion into the study.

This study was approved by the local Scientific and Ethics Committees of IRCCS* “Saverio de Bellis”*, Castellana Grotte (Ba), Italy, and it was part of a registered research on https://www.clinicaltrials.gov, reg. number: NCT01244945.

### 2.2. Symptom Assessment

Patients were evaluated with Gastrointestinal Symptom Scoring Rate (GSRS), a validated questionnaire for GI symptoms [21], and the Constipaq scoring system, a modified Constipation Scoring System (CSS) [22] (Table 1).

GSRS utilises a Likert scale, depending on intensity and frequency of GI symptoms experienced over the past 3 months according to a semiquantitative score where 1 meant absent, 2 meant mild, 3 meant moderate, and 4 meant severe and interfering with daily activities. The patients were asked to grade seven different symptoms: abdominal pain, borborygmi, bloating, flatulence, reduced frequency of evacuation, hard stools, and feeling of incomplete evacuation.

The Constipaq takes into account the score of specific constipation-related items and the score of the patient's quality of life (QoL) for constipation. Constipation is classified as mild (CSS score 6–10), moderate (CSS score 11–15), and severe (CSS score > 15).

The stool consistency was investigated using the Bristol stool form chart [23]. Constipation was also self-evaluated during the whole study period using a diary where the patients indicated the numbers of bowel movements, sense of incomplete evacuation (yes/no), straining at defecation (yes/no), number of ineffective defecation attempts, help for defecation (laxatives, suppositories, enema, and digitations), associated GI symptoms (mild, moderate, and severe), and impairment of daily activities.

### 2.3. Colonic Transit Time Calculation

Sixty radiopaque markers were divided into three tubes containing 20 markers each. The content of each tube was ingested with water at 12:00 pm for three consecutive days, and simple abdominal radiographs were taken at 12:00 pm on days 4 and 7, according to Metcalf et al. [24].

The sum of radiopaque markers was multiplied by 1.2 to obtain the value of colonic transit time (CTT) in hours. According to data on CTT of Western populations, the mean CTT value in healthy people is 30–40 hours [25]. Therefore, those patients with a CTT shorter than 40 hours were considered as having a normal transit time (NTC) [26]. Besides, following Metcalf's criteria, in order to diagnose STC, the patients had to show a total CTT longer than 68 hours or, alternatively, a segmental delayed CTT. In more detail, CTT was considered to be delayed in the right colon when it was longer than 25 hours; CTT in the left colon was delayed when it was longer than 31 hours; CTT in the rectum was delayed when it was longer than 32 hours [24].

### 2.4. Gut Peptides

Basal blood samples were obtained from participants in the study in the fasting state at least 12 hours after the last meal. Moreover, sequential blood withdrawals were obtained at 30, 60, 90, 120, 150, and 180 minutes after the supply of a test meal consisting of a ready-to-eat, gluten-free, and lactose-free 100 g muffin meal. The total energy intake was 378 kcal, with 57 g carbohydrates (61%), 14 g fats (33%), and 6 g proteins (6%). Blood samples were collected in ice chilled tubes containing 500 KIU/mL of aprotinin (100,000 KIU; MP Biomedicals, LLC, OH) and 1.0 mg of EDTA/mL blood. The separated plasma was stored at −70°C until assay. Plasma levels of neurotensin, motilin, CRF, and somatostatin were measured by enzyme immunoassay technique using commercial kits (Cloud-Clone Corp., TX, USA).

### 2.5. Genotyping

*NTS* rs1800832 A/G SNP was typed according to Vanakoski et al. [27]. In brief, PCR fragments were amplified with the following primers: 5′-GCTGAAGGAAAGAGGAAGTG-3′ and 5′-GGAGTAGCATGCATACAAGC-3′. PCR parameters were initial denaturation at 95°C for 2 minutes, followed by 30 cycles of 20 seconds at 95°C, 20 seconds at 56°C, 20 seconds at 72°C, and 5 minutes of final extension at 72°C. The amplicons were digested with the DdeI restriction enzyme and the products were separated by agarose gel electrophoresis. The rs6090453 C/G SNP in the* NTR1* gene was typed using the following primers: 5′-TCTCCAGGTGGTCTTCCTGT-3′ and 5′-GCAGAATCTTGGACCCTCAA-3′. PCR conditions were the same as those for the* NTS* rs1800832 A/G. The 214-bp amplicon was digested with the NsbI restriction enzyme and the products were separated by gel electrophoresis. In the presence of the G allele, the amplicon was restricted into the 111-bp and the 103-bp fragments.

### 2.6. Statistical Analysis

Unless otherwise specified, data were expressed as median and the 25th–75th interquartile range and nonparametric tests were performed in order to avoid violation of the assumption of the normal distribution. Data were evaluated by Mann–Whitney rank sum test or Kruskal-Wallis ANOVA test on ranks with Dunn's multiple comparison test where requested, unless otherwise indicated. The GI peptide concentrations at baseline and following the test meal administration were calculated as raw values and area under the curve (AUC) at seven time-points (0, 30, 60, 90, 120, 150, and 180 minutes) to yield an approximation of total release of each hormone over the time of observation.

The genotype frequencies of the analysed SNPs were estimated by gene counting. Statistical differences in genotypic distributions among the study groups were evaluated by *χ*^2^ test, as well as significant departures from Hardy-Weinberg equilibrium. Statistical significance was set at *p* < 0.05. A specific software package was used for the statistical analysis (StataCorp 2005, Stata Statistical Software: Release 9; College Station, TX, USA).

## 3. Results

### 3.1. Patients

**Figure 1:**
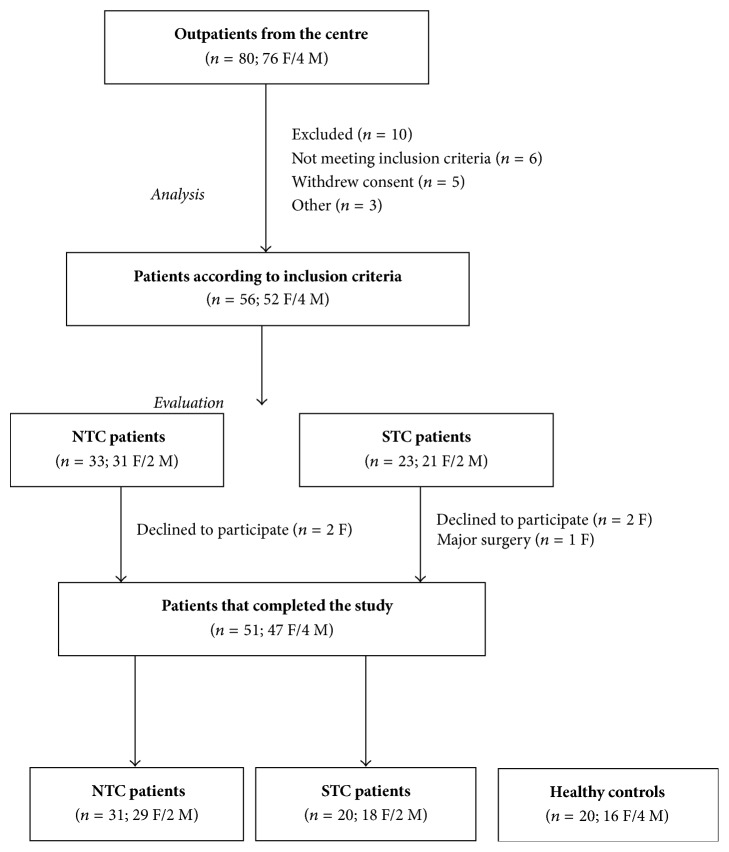
The flow of participants through the study.

Figure 1 depicts the flow of patients in the study. Overall 51 patients (47 F/4 M) were analysed: 31 (29 F/2 M) of them were diagnosed with NTC (total CTT = 18.0 hours, 10.0–38.9) and 20 (18 F/2 M) with STC (total CTT = 71.4 hours, 46.8–144.0). Sixteen STC patients had a delayed CTT in the left colon and 4 patients had a delayed CTT in both the left colon and rectum. No patient had an isolated delayed CTT in either the right colon or rectum. The CTT in the right colon was 12.6 hours (3.6–27.6 hours); the CTT in the left colon was 37.2 hours (31.2–124.8 hours); and the CTT in the rectum was 38.4 hours (36.0–67.2 hours). CTT values are expressed as median and the range. As HC group, 20 volunteers (16 F/4 M) were admitted to participate. At the start of the study, all the laboratory parameters were in the normal range (data not shown).

The age of the participants did not differ between HC and the groups of patients: HC (49.0 ± 11.3 yrs.), NTC (46.5 ± 11.4 yrs.), and STC (49.9 ± 12.7 yrs.) (*p* = ns; *t* test). Also, the body mass index in HC (25.1 ± 3.4 kg/m^2^), NTC patients (24.7 ± 3.3 kg/m^2^), and STC patients (24.1 ± 4.7 kg/m^2^) was not statistically different (*p* = ns; *t* test). Data are expressed as mean ± SD.

### 3.2. Symptom Assessment

Table 2 reports the median GSRS scores recorded in the NTC and STC patients. Both groups presented similar GSRS item scores, apart from* “reduced frequency of evacuation”* that was statistically higher in the STC group than in NTC one (*p* = 0.008, Mann–Whitney test).

Table 3 shows the median CSS and Constipaq score in NTC and STC patients. Both groups showed similar CCS items score, apart from CSS item C, stool frequency (number of defecations), that was statistically higher in the STC group than in NTC one (*p* = 0.048, Mann–Whitney test). Oppositely, the CSS total score and Constipaq score were not different in STC patients from those in NTC patients and both groups, with a median CSS score >15, which showed a severe constipation level.

### 3.3. Plasma Gut Peptide Concentrations

**Figure 2:**
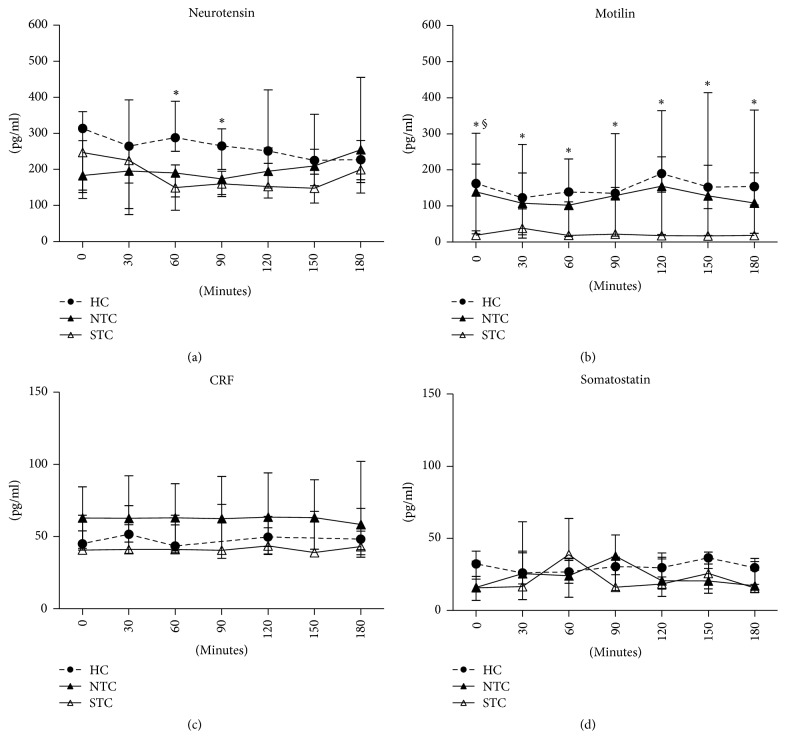
The figure plots the serial changes in the GI peptide concentrations in healthy controls (HC) and constipated patients with normal transit constipation (NTC) and slow transit constipation (STC) recorded at baseline and every 30 minutes up to 180 minutes following meal administration. Data reported as median and the 25th–75th percentile. Each sample time was analysed by Kruskal-Wallis and Dunn's multiple comparison test (^*∗*^STC versus HC, ^§^STC versus NTC, *p* < 0.05).

Figure 2 plots the GI peptide concentrations in STC and NTC patients as well as in HC recorded at baseline and every 30 minutes up to 180 minutes after meal administration.

Neurotensin concentrations in STC patients were lower compared to those in HC with significant differences at 60 minutes (Kruskal-Wallis *p* = 0.005, Dunn's multiple comparison test *p* < 0.05) and 90 minutes (Kruskal-Wallis *p* = 0.01, Dunn's multiple comparison test *p* < 0.05) (Figure 2(a)).

Motilin release was markedly and significantly different among the groups, showing the lowest baseline concentration in STC patients compared to both NTC and HC (Kruskal-Wallis *p* = 0.0019, Dunn's multiple comparison test *p* < 0.05). The difference persisted significantly between STC patients and HC in the whole postprandial times of observation (Kruskal-Wallis *p* < 0.05, Dunn's multiple comparison test *p* < 0.05) although, throughout the curve, STC patients showed 85% and 82% lower levels than HC and NTC, respectively (Figure 2(b)).

Lastly, both CRF and somatostatin concentrations were not different among groups at any time during the analysis (Figures 2(c) and 2(d)).

To evaluate the total release of GI peptides over the time of observation, AUCs of gut peptides were analysed in the three groups. Significant differences were found with regard to the AUC of neurotensin (*p* = 0.0128, Kruskal-Wallis ANOVA test; STC versus HC *p* < 0.05, Dunn's multiple comparison test) and motilin (*p* = 0.0093, Kruskal-Wallis ANOVA test; STC versus HC, *p* < 0.05, Dunn's multiple comparison test). No differences were found as concerns the AUCs of CRF and somatostatin (Table 4). Finally, no significant correlation between serum levels of motilin and neurotensin as well as between the symptom profile and gut peptide levels was found (data not shown).

### 3.4. *NTS* and* NTR1* Polymorphisms Analyses

Neurotensin system genetics was investigated.  Table 5 reports the genotype frequencies of the* NTS* rs1800832 A/G and the* NTSR1* rs6090453 C/G SNPs in the three groups. As concerns the* NTS* polymorphism, observed genotype frequencies conformed to the Hardy-Weinberg equilibrium, either when the total sample was tested (*p* = 0.4792, *χ*^2^ test) and when the groups were investigated separately (*p* = 0.4792, HC; *p* = 0.3444, NTC; and *p* = 0.8139, STC; *χ*^2^ test). The frequency distribution of the genotypes was statistically different among the different groups (*p* = 0.0145, *χ*^2^ test). Neurotensin concentrations were analysed according to the A/G SNP. Subjects carrying the G allele showed 29% lower neurotensin levels compared to those who do not carry this allele: plasma concentrations were 156.3 and 106.4–200.4 and 219.3 and 147.0–290.1 pg/ml, in AG and AA genotypes carrying subjects, respectively (*p* = 0.0205, Mann–Whitney test).

The rs6090453 C/G SNP in the* NTSR1* gene was analysed. Observed genotype frequencies conformed to the Hardy-Weinberg equilibrium (*p* = 0.4589, total sample; *p* = 0.6583, HC; *p* = 0.5725, NTC; *p* = 0.6458, STC; *χ*^2^ test). No statistically significant differences were present in the genotype frequencies (*p* = 0.7583, *χ*^2^ test) among the three groups of subjects. According to the* NTSR1* polymorphism, neurotensin concentrations were 242.2 and 164.2–314.9, 186.1 and 145.2–258.1, and 188.8 and 138.7–320.1 pg/ml in CC, CG, and GG subjects, respectively, without statistical differences among groups (*p* = 0.2544, Kruskal-Wallis ANOVA test).

## 4. Discussion

Functional constipation may be caused by different factors and it is differently perceived by patients. This study was firstly aimed at investigating possible differences in the symptom profiles of NTC and STC patients. Clinically, functional constipation can be evaluated by using specific GI questionnaires (i.e., GSRS and Constipaq). However, these tools are mainly used as inclusion criteria for clinical trials and to support epidemiological studies, and their application is indeed infrequent in the clinical practice [28]. Based on the present results, the administration of dedicated symptomatic questionnaires provides little, if any, utility in discriminating STC with respect to NTC patients due to the high degree of symptom overlap between the two groups. The only symptom highlighted by both the questionnaires was the frequency of evacuation, thus confirming that general or constipation-related symptoms/signs cannot clearly identify the underneath pathophysiology of constipation.

As a whole, constipation accounts for about 9% of patients attending a tertiary referral Italian Colorectal Unit and about 36% of them suffer from STC [29], suggesting a putative etiological role for some GI peptides. Thus, the second aim of the study was to evaluate the profiles of motilin, neurotensin, CRF, and somatostatin in NTC and STC patients in comparison to healthy subjects. All these peptides are involved in alterations of the colonic motility [8, 9] and in some cases their agonist/antagonist have already been tested as treatment options in functional constipation [30].

In the present study, healthy subjects and severe constipated patients, categorized according to the colonic transit time, showed a dramatic difference in motilin serum profiles. Both basal motilin and the whole postprandial profile were found to be significantly reduced in STC with 85% and 82% lower levels than HC and NTC ones, respectively. The analysis of motilin AUC confirmed the significant difference between STC and HC. These findings agree with the peculiar properties of motilin on GI motility. In the upper gut, this peptide is the most important factor in controlling the interdigestive migrating motor complex (MMC) [31], and motilin receptor agonists have been proposed for treating gastroparesis or conditions with slow gastric emptying [32]. Besides, the motilin receptor agonists are supposed to affect also movements of isolated colon or intact colon in patients with functional constipation. No clear proof supports this evidence, even if motilin receptors have been found in the muscle and myenteric plexus of the human colon [33]. Our data are consistent with early information about motilin and constipation. In 1986, Sjölund et al. [34] investigated the plasma motilin levels in subjects with long-standing severe functional constipation irrespective of colonic transit time, showing lower basal motilin concentrations and lower release after the test meal than healthy subjects. Peracchi et al. [35] obtained similar results in women with idiopathic STC compared to healthy ones, showing no increase in the postprandial motilin levels as well as cholecystokinin, neurotensin, and somatostatin. Penning et al. [7] evaluated both proximal and distal GI hormones in fasting state and postprandial state in STC patients, highlighting that the proximal gut GI hormones (cholecystokinin, gastrin, and pancreatic polypeptide) apart from motilin increased, whereas the distal gut ones (neurotensin and polypeptide YY) decreased. In children, a recent paper described lower serum motilin levels in paediatric constipated patients than in HC, but without evaluating the colonic transit time. Furthermore, serum motilin levels correlated with Bristol score in the whole group but not in constipated children [10]. With respect to these previous reports, our study included patients of both genders with severe constipation and put in evidence a peculiar motilin behaviour in STC, and overall it let us hypothesize that the assessment of motilin levels could be useful for the STC diagnosis, in addition to representing a pathophysiological marker of GI motility.

As concerns neurotensin, its circulating concentrations were significantly lower in STC than HC, particularly in the early postprandial period, thus confirming the altered motility in this group of patients. Neurotensin has recently been shown to be a neurotransmitter, acting either via the nonadrenergic noncholinergic (NANC) excitatory nerves of the human GI tract or directly on the colonic smooth muscle. A decrease of neurotensin-induced contractions has already been observed in vitro in colon segments resected from STC patients and this evidence suggests that neurotensin plays an important role in the dysmotility pattern in such patients [36]. A study on the motor patterns occurring in the rat intact colon put in evidence that low neurotensin concentrations inhibit the propagating long distance contractions and rhythmic propagating motor complexes; in its place a slow propagating rhythmic segmental motor pattern occurs. Moreover, high concentrations of neurotensin are capable of restoring long distance contraction activity and inhibiting the segmental activity [37]. If confirmed in humans, this behaviour of neurotensin could explain the physiological background of colonic slow transit time in some patients with constipation. However, in addition to the stimulation of gut motility, neurotensin plays a number of roles in the GI disorders. Not only was it shown as being implicated in intestinal inflammation, as demonstrated by its increased peripheral levels found in paediatric celiac patients [38], but also a profound opioid-independent analgesic effect was demonstrated [14]. This finding leads to interesting pathophysiological implications for symptoms of constipation.

Given the putative role of this peptide as neurotransmitter in the enteric nervous system, a part for neurotensin genetics in modifying the risk of functional constipation could be also hypothesized. In search for causative polymorphisms associated with the familial clustering of the disease, two SNPs of the neurotensin system genes (the rs1800832 A/G in the 5′ UTR of the* NTS* gene and the rs6090453 C/G intron variant in the* NTSR1*) were investigated. As regards the* NTS* rs1800832 SNP, none of the subjects under investigation showed being a carrier of two copies of the minor G allele, according to the reported very low frequency of this variant [39]. Of note, as already reported by our group [19], the G variant could be considered functional since it could affect the efficiency of the translation, being located in the conserved Kozak motif of the gene [40], which identifies the initiator AUG. Indeed, when fasting neurotensin concentrations were analysed according to this polymorphism, subjects carrying the G allele showed about 30% lower neurotensin levels than the AA homozygotes. The frequency distribution of genotypes was significantly different among the three study groups: the NTC group, the one showing the significantly lowest basal concentration of the peptide, showed also the highest prevalence of heterozygotes subjects. Therefore, it appears that the* NTS *genetics could influence neurotensin levels, without affecting the pattern of gut motility, possibly being associated with the symptom profile of constipation [13]. No differences among the three groups were found concerning the* NTSR1* polymorphism, with the frequency distribution of allelic variants resembling that already reported [41].

As for CRF, its circulating concentrations were similar in HC and constipated patients, regardless of colonic transit time. CRF is thought to be involved in autonomic dysfunction. Low vagal activity can lead to a reduction in bowel contractions, reduced motility, and constipation, as reported in female patients suffering from IBS with constipation [42]. Assuming the close association between the stress axis and the autonomic nervous system, increased sympathetic tone recorded in constipation has been related to the increase in CRF expression [43] and it has been reported that subjects with functional constipation have CRF patterns different from those in IBS patients [44]. Our study showed lower CRF levels in STC than NTC patients, but without a significant difference. Probably, this peptide is not directly involved in functional constipation; thus the evaluation of its circulating concentrations seems not to be able either to provide useful information on pathophysiology of constipation or to be used as a putative diagnostic marker.

Finally, as regards somatostatin no differences were present in constipated patients according to the colon transit time and HC. Actually, long lasting somatostatin analogues (octreotide, lanreotide) seem to be limited to the treatment of neuroendocrine tumours and adjuvant treatment of oesophageal variceal bleeding and pancreatic fistulas [45], and some authors found that octreotide was not able to affect colonic motility in paediatric patients with functional constipation [46].

## 5. Conclusions

In conclusion, patients with severe functional constipation have specific alterations in the circulating profile of some GI peptides, particularly neurotensin and motilin, linked to impaired colonic motility. Besides, the study of the genetics of neurotensin appears to be an appropriate target for investigating the multifactorial bases of functional constipation. All these findings may be of pathophysiological significance and may help in further characterizing STC patients.

## Figures and Tables

**Table 1:** CSS system code and Constipaq code.

*C stool frequency (number of defecations) *	
(0) >2 per week	
(1) 2 per week	
(2) 1 per week	
(3) <1 per week	
(4) <1 per month	
*N incomplete defecation *	
(0) Never	
(1) <1 per month	
(2) 1–4 per month	
(3) 1-2 per week	
(4) >2 per week	
*T time spent in toilet (minutes) *	
(0) <5	
(1) 5–10	
(2) 10–20	
(3) 20–30	
(4) >30	
*A unfruitful attempts (numbers) *	
(0) Never	
(1) 1–3 per day	
(2) 4–6 per day	
(3) 7–9 per day	
(4) >9 per day	
*O obstruction, pain, straining*	
(0) Never	
(1) <1 per month	
(2) 1–4 per month	
(3) 1-2 per week	
(4) >2 per week	
*S abdominal discomfort, pain, bloating*	
(0) Never	
(1) <1 per month	
(2) 1–4 per month	
(3) 1-2 per week	
(4) >2 per week	
*I-P helping in defecation*	
(0) <1 per week	
(1) Laxatives, suppositories (*I*) > 1 per week	
(2) Enema, digitation (*P*) > 1 per week	
*Q duration of constipation (year)*	
(0) <1	
(1) 1–5	
(2) 6–10	
(3) 11–20	
(4) >20	

Constipaq = CCS (0–30) + number of capital letters (0–9) + QoL (0–3) ×  *n*.

**Table 2:** GSRS symptom scores recorded in constipated patients categorized according to the time of colonic transit.

	NTC (31)	STC (20)	*p*
Abdominal pain	2 (2-3)	2 (1–3)	ns
Borborygmi	2 (2-3)	2 (2-3)	ns
Bloating	3 (2–4)	3 (2-3)	ns
Flatulence	2 (2-3)	2 (2-3)	ns
Reduced frequency of evacuation	2 (1-2)	3 (2-3)	0.008
Hard stools	3 (2–4)	3.5 (2–4)	ns
Feeling of incomplete evacuation	3 (3-4)	3 (3-4)	ns

Data expressed as median and the 25th–75th interquartile range and analysed by Mann–Whitney test. NTC: normal transit constipation; STC: slow transit constipation.

**Table 3:** CSS data in constipated patients categorized according to the time of colonic transit.

Parameters	NTC (31)	STC (20)	CSS scale
*C* stool frequency	0 (0-1)	1 (0–2)	0: >2*; 1*:* 2 per week *(*p* = 0.048)
*N* incomplete defecation	3 (2–4)	4 (2–4)	*3*:* 1-2 per week *(*p* = ns)
*T* time spent in toilet (min)	1 (1-2)	1 (1-2)	*1*:* 5*–*10 min *(*p* = ns)
*A* unfruitful attempts (*n*)	1 (0-1)	1 (1-1)	*1*:* 1*–*3 per day *(*p* = ns)
*O* obstruction, pain, strain	3 (3-4)	3 (2–4)	*3*:* 1-2 per week *(*p* = ns)
*S* abdominal discomfort, pain, bloating	3 (3-4)	3 (2–4)	*3*:* 1-2 per week *(*p* = ns)
*I* + *P* help for defecation	1 (0-1)	1 (0–2)	*Laxative suppositories *≥* 1 per week *(*p* = ns)
*Q* duration of constipation (year)	3 (2–4)	4 (2–4)	*3*:* 11*–*20 years *(*p* = ns)
*CSS score* (CSS *p* + number of capital letters)	24 (20–25)	24 (19–28)	*>15 score*:* severe constipation *(*p* = ns)
*Constipaq score*	58 (49–72)	61 (48–72)	(*p* = ns)

Data expressed as median and the 25th–75th interquartile range and analysed by Mann–Whitney test. NTC: normal transit constipation; STC: slow transit constipation.

**Table 4:** AUC of gut peptides release in healthy controls and constipated patients categorized according to the time of colonic transit.

	HC (20)	NTC (31)	STC (20)	*p*
Neurotensin	1656.0^a^ (1320.0–2152.0)	1141.0^ab^ (982.3–1373.0)	1124.0^b^ (654.0–1532.0)	0.0128
Motilin	950.3^a^ (731.8–1252.0)	879.7^ab^ (169.3–1913.0)	160.0^b^ (81.75–183.0)	0.0093
CRF	290.7^a^ (265.9–317.2)	369.7^a^ (235.9–549.9)	246.2^a^ (229.0–407.7)	ns
Somatostatin	185.6^a^ (141.0–213.1)	147.5^a^ (76.83–213.8)	161.8^a^ (103.5–255.2)	ns

Data expressed as median and the 25th–75th interquartile range and analysed by Kruskal-Wallis ANOVA with Dunn's multiple comparison test. Values not sharing a common superscript differ significantly. HC: healthy controls; NTC: normal transit constipation; STC: slow transit constipation.

**Table 5:** Genotypes frequency distributions of the neurotensin system SNPs in healthy controls and constipated patients categorized according to the time of colonic transit.

	*NTS *rs1800832 A/G		*NTSR1 *rs6090453 C/G		
	*p* = 0.0145		*p* = 0.7583		
	AA	AG	CC	CG	GG
HC (*n* = 20)	20 (100%)	0 (0%)	8 (40%)	10 (50%)	2 (10%)
NTC (*n* = 31)	22 (71%)	9 (29%)	8 (26%)	17 (55%)	6 (19%)
STC (*n* = 20)	18 (90%)	2 (10%)	5 (25%)	11 (55%)	4 (20%)

Absolute numbers and frequencies (in parentheses). *p*: *χ*^2^ test. HC: healthy controls; NTC: normal transit constipation; STC: slow transit constipation.

## Abbreviations

AUC: Area under the curve
CRF: Corticotrophin-releasing factor
CSS: Constipation Scoring System
CTT: Colonic transit time
GI: Gastrointestinal
GSRS: Gastrointestinal Symptom Scoring Rate
HC: Healthy controls
IBS: Irritable bowel syndrome
MMC: Migrating motor complex
NANC: Nonadrenergic noncholinergic
NTC: Normal transit constipation
*NTR1*: Neurotensin Receptor 1
*NTS*: Proneurotensin gene
STC: Slow transit constipation
QoL: Quality of life
UTR: Untranslated region.
